# Solid electrolytes for practical all-solid-state lithium batteries: integrating ion transport, interfacial mechanics, and scalable manufacturing

**DOI:** 10.3389/fchem.2026.1964251

**Published:** 2026-09-18

**Authors:** Xianzheng Liu, Nashrah Hani Jamadon, Wenlian Li, Tongtong Dong, Yuan Li, Xiujun Yuan

**Affiliations:** 1 College of Mechanical Engineering, Shandong Huayu University of Technology, Dezhou, Shandong, China; 2 Department of Mechanical and Manufacturing Engineering, Faculty of Engineering and Built Environment, Universiti Kebangsaan Malaysia, Bangi, Selangor, Malaysia

**Keywords:** all-solid-state lithium batteries, interface mechanics, lithium dendrite, low stack pressure, scalable manufacturing, solid electrolyte

## Abstract

All-solid-state lithium batteries are increasingly viewed as a route toward safer and higher-energy rechargeable batteries, yet the transition from highly conductive electrolyte materials to practically competitive cells remains difficult. The central challenge is no longer simply to maximize bulk ionic conductivity. Solid electrolytes must simultaneously provide continuous ion-transport pathways, stable electrode contact, limited electronic leakage, sufficient mechanical compliance or fracture resistance, and compatibility with manufacturing processes that produce thin electrolyte layers and high-loading electrodes. This review examines solid electrolytes from this integrated materials–mechanics perspective. Oxide, sulfide, halide, nitride, polymer, and composite electrolytes are compared according to the relationship between transport behavior, chemical stability, mechanical response, interface evolution, and processability. Particular attention is given to contact loss, interphase formation, void generation, lithium penetration, and the role of external stack pressure. Recent progress in low-temperature ceramic processing, fluorinated sulfides and halides, structured composite electrolytes, and advanced polymer electrolytes illustrates how these traditionally coupled limitations can be partly decoupled. Manufacturing strategies including dry processing, thin electrolyte fabrication, melt infiltration, and pressure-efficient cell architectures are further discussed. Finally, practical electrolyte design is proposed to shift from optimizing isolated material properties toward balancing conductivity, thickness, interfacial stability, mechanical reliability, and scalable processing under realistic cell conditions.

## 1 Introduction

The replacement of flammable liquid electrolytes by solid ion conductors has been one of the most persistent directions in advanced lithium-battery research. In principle, all-solid-state lithium batteries (ASSLBs) offer several attractive possibilities: improved thermal safety, compatibility with lithium-metal or high-capacity alloy anodes, simplified separator functions, and access to high-voltage positive electrodes. These advantages have stimulated intensive research into inorganic ceramics, sulfide glasses and crystals, halides, polymers, and multiphase composite electrolytes (Chan et al.,2024; Liu X. et al., 2026). Nevertheless, a persistent gap remains between electrolyte properties measured on dense laboratory specimens and the performance obtained in practical cells.

Early electrolyte development was dominated by the search for high room-temperature ionic conductivity. This objective produced major milestones such as garnet-type Li_7_La_3_Zr_2_O_12_ (LLZO) (Murugan et al., 2007; Yoon et al., 2023), Li_10_GeP_2_S_12_ (LGPS) (Kamaya et al., 2011), highly conductive sulfide superionic conductors (Kato et al., 2016), and chloride-based electrolytes suitable for high-voltage positive electrodes (Asano et al., 2018). Yet conductivity alone does not determine the resistance of a functioning solid-state cell. Ion transport also depends on electrolyte thickness, grain boundaries, electrode tortuosity, real interfacial contact area, and the evolution of mechanically and chemically heterogeneous interphases (Banerjee et al., 2020; Cao et al., 2025).

This distinction becomes increasingly important as ASSLB research moves toward high areal capacity, thin separators and lithium-metal anodes. A ceramic with excellent bulk conductivity may generate large interfacial resistance because its rigid surface cannot conform to electrode particles. A soft sulfide may establish intimate contact but undergo chemical degradation or mechanical deformation. A polymer can accommodate electrode-volume changes but may provide insufficient room-temperature conductivity or oxidative stability. Moreover, lithium penetration is now recognized as a coupled electrochemical and mechanical process rather than a phenomenon that can be prevented simply by selecting a stiff electrolyte (Hatzell et al., 2020; Ning et al., 2023).

Accordingly, this review evaluates solid electrolytes through a practical engineering framework. Instead of ranking electrolyte families by conductivity alone, emphasis is placed on the coupling among ion transport, interfacial chemistry, mechanical response, pressure requirement, and manufacturability. This perspective is particularly relevant to the next stage of ASSLB development, where cell-level performance must increasingly be demonstrated using thin electrolytes, high-loading electrodes, and moderate stack pressure rather than favorable laboratory conditions. The overall framework of this review, linking electrolyte chemistry with ion transport, interfacial chemo-mechanics, low-pressure operation, and scalable manufacturing toward practical ASSLBs, is schematically illustrated in Figure 1.

**FIGURE 1:**
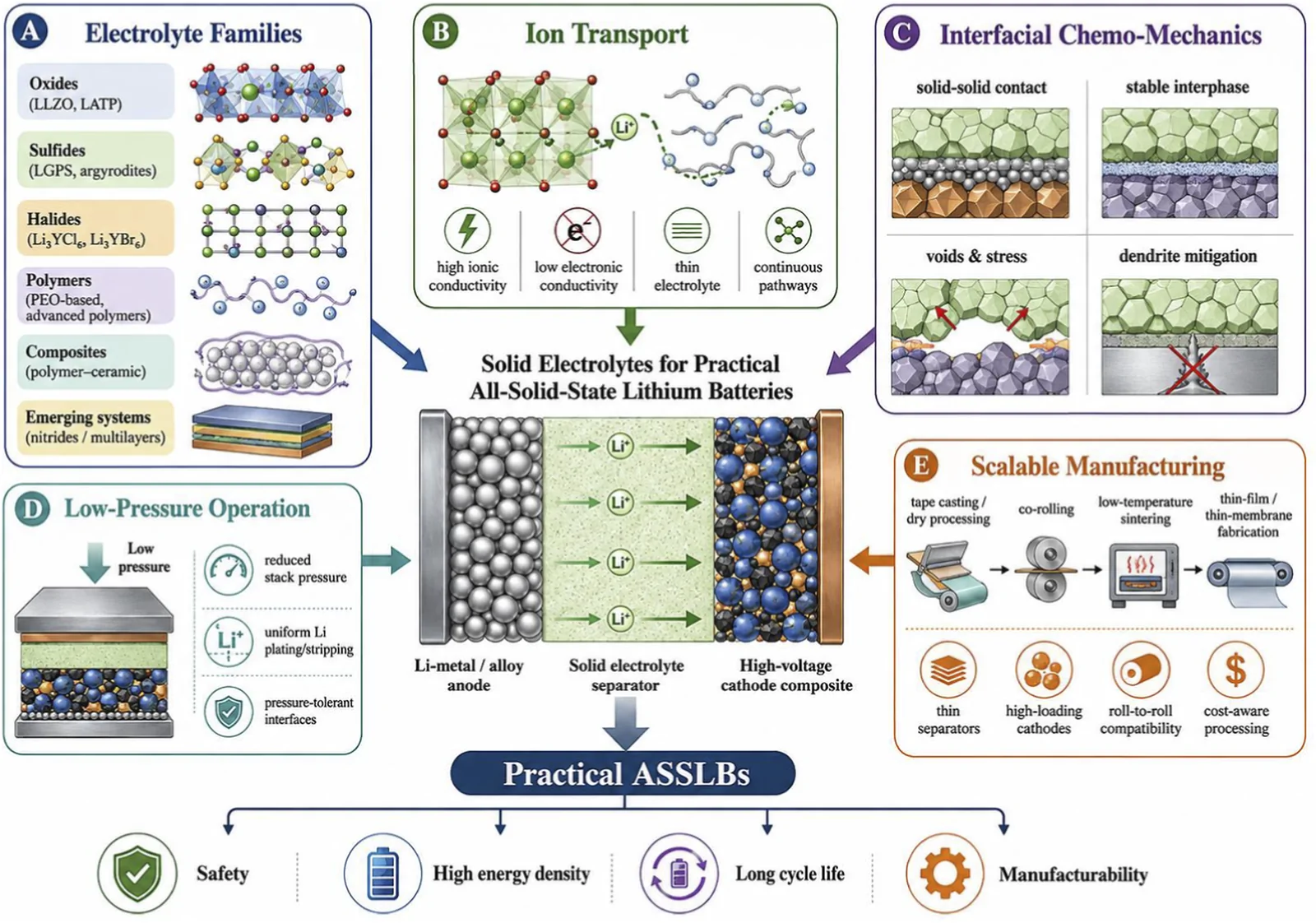
Schematic overview of solid electrolyte design for practical ASSLBs. **(A)** Major electrolyte families. **(B)** Key ion-transport requirements. **(C)** Interfacial chemo-mechanics, including contact stability, void/stress control, and dendrite mitigation. **(D)** Low-pressure operation with pressure-tolerant interfaces and uniform Li plating/stripping. **(E)** Scalable manufacturing strategies. Coordinated optimization of these factors enables practical ASSLBs with improved safety, energy density, cycle life, and manufacturability.

## 2 Beyond bulk conductivity: coupled requirements for solid electrolytes

Ionic conductivity remains a fundamental electrolyte parameter because insufficient Li-ion mobility produces polarization and restricts power capability. However, the resistance contribution of an electrolyte layer depends approximately on thickness divided by conductivity. Consequently, reducing a separator from several hundred micrometers to tens of micrometers can be as important for cell resistance and specific energy as improving intrinsic conductivity. This consideration explains why manufacturability and mechanical integrity of thin membranes have become central electrolyte characteristics.

The second requirement is negligible electronic conductivity. Solid electrolytes are normally treated as purely ionic conductors, yet small electronic currents can become important during long-term lithium plating. Han et al. (2019) demonstrated that electronic conduction within solid electrolytes can facilitate internal lithium nucleation, illustrating why a mechanically strong material alone does not guarantee resistance to short circuiting. Current focusing around pores, grain boundaries and surface defects further couples electronic leakage to local electrochemical deposition.

Mechanical properties introduce another trade-off. High modulus is useful for maintaining structural integrity but does not necessarily create stable contact. Rigid ceramics contact rough composite electrodes over only a fraction of their nominal area, generating high local current density. During cycling, electrode particles change volume, interphases grow, and lithium is repeatedly stripped and replated. These processes alter stress and contact conditions. Operando tomography has shown that void formation and interphase evolution are directly linked to electrochemical behavior at buried solid-state interfaces (Lewis et al., 2021).

The original material-selection problem must therefore be expanded. An effective electrolyte should combine sufficiently high conductivity with low electronic leakage, chemical compatibility, controllable interphase chemistry, resistance to fracture, and enough deformability or engineered interfacial compliance to preserve contact. This multidimensional requirement explains why no single electrolyte family currently dominates every practical ASSLB configuration.

### 2.1 Sources of performance variability and data comparability

Reported properties of a nominally identical solid-electrolyte family can vary substantially among studies and should therefore not be interpreted as fixed material constants. For inorganic electrolytes, ionic conductivity is strongly affected by phase purity, relative density, grain size, grain-boundary chemistry, defect concentration, particle size, and thermal or mechanical processing history. Sulfide electrolytes are additionally sensitive to compaction pressure, moisture exposure, particle morphology, and mixing procedures, whereas polymer electrolytes are influenced by molecular weight, salt concentration, crystallinity, plasticizer content, and thermal history. Composite electrolytes introduce further variables associated with filler loading, dispersion, orientation, percolation, and polymer–ceramic interfacial chemistry. Even electrode-processing variables can modify the apparent cell performance; for example, the type and incorporation route of conductive agents during sulfide-electrolyte coating can change coating morphology, electrochemically active area, and electronic percolation within composite cathodes (Kim et al., 2025).

Measurement conditions introduce a second level of variability. Ionic conductivity depends on temperature, electrode configuration, pellet thickness, density, impedance-fitting procedure, and thermal equilibration, while reported mechanical properties depend strongly on porosity and whether tensile testing, compression, nanoindentation, or other methods are used. At the cell level, electrolyte thickness, cathode areal loading, active-material fraction, current density, voltage window, stack pressure, temperature, and pressure-control mode can dominate apparent cycle life. Consequently, cross-study comparison should preferably combine intrinsic material descriptors with cell-level parameters rather than rank electrolytes using conductivity or cycle number alone. Phase purity, electrolyte density and thickness, measurement temperature, electrode loading, applied pressure, and cycling protocol should therefore be regarded as minimum reporting parameters for meaningful engineering comparison.

## 3 Major electrolyte families through an engineering perspective

### 3.1 Oxide electrolytes: stability versus rigidity

Oxide electrolytes are attractive because of their thermal stability, relatively broad electrochemical operating range and robustness during handling. Garnet-type LLZO is particularly important because it combines lithium-metal compatibility with room-temperature ionic conductivity in the 10^−4^–10^–3^ S cm^-1^ range after suitable compositional and microstructural control (Murugan et al., 2007). Oxides also include NASICON-type compounds and perovskite conductors, although some Ti-containing compositions are unstable in direct contact with lithium.

The principal disadvantage of dense oxide ceramics is mechanical. High elastic modulus and limited plastic deformation make intimate electrode contact difficult (Yu et al., 2016). Surface contamination, including carbonate formation on LLZO, may further increase interface resistance. Conventional garnet processing also frequently requires temperatures approaching or exceeding 1000 °C, introducing lithium loss, compositional deviation, energy consumption and difficulties in co-processing electrolyte and electrode layers (Balaish et al., 2025).

Recent processing strategies suggest that these restrictions are not intrinsic to the garnet chemistry. Kwon et al. (2025) introduced a disorder-driven approach in which mechanically activated amorphous precursors were densified before crystallization. Cubic garnet formation began at approximately 350 °C, and a single 500 °C treatment produced an electrolyte with a room-temperature conductivity of 1.8 × 10^−4^ S cm^-1^, substantially reducing the thermal requirement compared with conventional sintering. Such results are important not only because of conductivity, but because lower-temperature processing may enable thinner membranes, less severe interfacial reactions and more economical production.

Oxide development should therefore increasingly focus on microstructural connectivity and interface mechanics rather than pursuing bulk conductivity in isolation. Surface modification, compliant interlayers, low-temperature densification and defect-controlled processing are likely to be particularly valuable (Liu Y. et al., 2026).

### 3.2 Sulfide electrolytes: conductivity and processability with interfacial constraints

Sulfide solid electrolytes represent the opposite mechanical extreme. Their weaker bonding generally provides higher deformability than oxide ceramics, allowing powders to form dense ion-conducting contacts by cold pressing (Torii et al., 2025). LGPS and related materials demonstrated that solid-state Li-ion conductivity can approach or exceed that of conventional liquid electrolytes (Kamaya et al., 2011). Subsequent sulfide conductors have reached conductivities in the mS cm^-1^ to tens-of-mS cm^-1^ regime, enabling high-rate ASSLB operation (Kato et al., 2016).

The softness of sulfides is advantageous in composite cathodes because electrolyte particles can better conform to active-material surfaces. High-loading silicon electrodes have also been successfully coupled with sulfide electrolytes, demonstrating the possibility of eliminating conventional graphite and excess conductive carbon while maintaining large reversible capacity (Tan et al., 2021). However, mechanical compliance does not eliminate interface degradation. Sulfides can react with lithium metal and high-voltage cathodes, and many compositions are moisture sensitive. Electrochemical decomposition may produce mixed-conducting interphases that continuously propagate instead of forming self-limiting passivation layers.

However, intimate mechanical contact alone does not guarantee an optimized composite electrode. The type and incorporation route of conductive agents during sulfide-electrolyte coating can substantially modify coating morphology, electrochemically accessible surface area and electronic percolation, highlighting the importance of electrode-processing protocols in addition to electrolyte chemistry (Kim et al., 2025).

Fluorination is emerging as one route to modify both bulk and interfacial behavior. A 2026 study employed bulk-to-interface fluorination to stabilize a sulfide-based lithium-metal system. The resulting pouch cells operated at a stack pressure of only 2.5 MPa for more than 350 cycles at 1 C with 85% capacity retention; the authors also reported specific energy above 400 Wh kg^-1^ when calculated from the solid electrolyte, lithium and positive-electrode components (Sang et al., 2026). This is significant because conventional sulfide cells often rely on considerably higher mechanical pressure to preserve contact.

Sulfide research is therefore shifting from conductivity optimization toward controlled interphase chemistry, air tolerance, pressure reduction and continuous manufacturing. The ability to densify sulfide films without high-temperature sintering makes them particularly compatible with dry electrode and roll-processing concepts.

### 3.3 Halide electrolytes

Halide electrolytes have developed rapidly because they combine comparatively soft mechanical behavior with favorable oxidative stability against high-voltage positive electrodes (Gong et al., 2025). The early demonstration of Li_3_YCl_6_ and Li_3_YBr_6_ established halides as practical solid ion conductors for approximately 4 V-class cathodes (Asano et al., 2018). Their relatively weak Li–halogen interactions and structurally disordered migration networks provide opportunities for further conductivity enhancement.

Recent work has shifted from simple compositional substitution toward controlling collective lattice dynamics. Liu et al. (2024) demonstrated that tuning collective anion motion in halide electrolytes can generate superionic transport, with optimized compositions reaching conductivities on the order of several to approximately 10 mS cm^-1^. This finding reinforces the idea that ion transport is governed not only by static vacancy concentration but also by dynamic lattice response.

Chemical stability remains asymmetric: many halides tolerate high positive-electrode potentials but are readily reduced at lithium metal. Interface layers or hybrid electrolyte architectures are therefore commonly required. New fluorination strategies attempt to address this issue while maintaining conductivity. Li et al. (2026) developed heavily fluorinated halide/sulfide ion conductors using an anion-exchange route, with LiF-rich interphases contributing to enhanced oxidative stability.

### 3.4 Nitride electrolytes and multilayer/gradient architectures

Nitride electrolytes are emerging as specialized components for lithium-metal interfaces rather than universal replacements for oxide, sulfide, or halide separators. Vacancy-rich β-Li_3_N provides a representative example. Li et al. (2025) reported an ionic conductivity of 2.14 × 10^−3^ S cm^-1^ at 25 °C together with an electronic conductivity of approximately 4.5 × 10^−10^ S cm^-1^. Lithium symmetric cells exhibited high current-density tolerance and prolonged stripping/plating stability. Importantly, the strongest full-cell demonstrations employed β-Li_3_N as a Li-compatible interfacial electrolyte in combination with halide electrolytes rather than as the sole separator. This configuration illustrates the principal engineering role of nitrides: providing rapid Li-ion transport and chemical compatibility near lithium metal while another electrolyte phase supplies oxidative stability toward the cathode. Remaining challenges include precise vacancy and stoichiometry control, large-area thin-film fabrication, compatibility with high-voltage cathodes, and maintaining stable interfaces during prolonged processing and cycling.

Multilayer and gradient electrolytes extend this concept by spatially separating functions that are difficult to satisfy within a chemically homogeneous material. A compliant Li-facing layer can preserve contact during stripping and plating, whereas a mechanically stronger or more oxidation-resistant cathode-facing region can stabilize high-voltage interfaces. Recent gradient architectures have further replaced abrupt internal interfaces with continuous composition and mechanical-property transitions. For example, a mechano-integrated LATP/polymer gradient electrolyte reported in 2026 used a membrane of approximately 40 μm, combined spatially graded ceramic loading with a common polymer framework, and achieved prolonged Li plating/stripping for more than 7500 h while also enabling stack-pressure-free NCM811 pouch-cell operation (Yi et al., 2026). The key bottlenecks for these architectures are no longer only ionic conductivity, but also resistance across internal interfaces, delamination, chemical interdiffusion, thickness penalties, reproducibility of the gradient, and compatibility with continuous coating or roll-to-roll manufacturing.

### 3.5 Polymer and composite electrolytes

Polymer electrolytes provide a fundamentally different route to interface stabilization. Their low modulus allows conformal contact and tolerance of electrode-volume change, and solution casting, extrusion and coating are generally more compatible with large-area manufacturing than ceramic sintering. The classic limitation is that ion transport in common poly (ethylene oxide)-based systems is strongly coupled to segmental motion. Crystallinity therefore suppresses room-temperature conductivity, while the oxidative stability of ether-based polymers restricts direct coupling with high-voltage cathodes.

Recent assessments of polymer/NCM interfaces further emphasize that practical high-voltage compatibility is controlled jointly by polymer molecular chemistry, cathode-surface reactivity and maintenance of physical contact. Molecular regulation of the polymer, cathode coatings and *in situ* polymerization have therefore emerged as complementary approaches for suppressing interfacial reactions and resistance growth (Lin et al., 2025).

Several recent approaches aim to escape this transport–stability trade-off. Local-high-concentration polymer environments can alter Li-ion coordination while maintaining a mechanically continuous matrix. Zhang et al. (2024) developed a single-phase local-high-concentration solid polymer electrolyte that simultaneously improved interfacial behavior and high-voltage cell operation. Molecular architecture provides another route. Chen et al. (2025) reported a nonflammable polymer electrolyte produced through puzzle-like molecular assembly, achieving an ionic conductivity of 0.432 mS cm^-1^, a Li-ion transference number of 0.70 and an oxidative stability of approximately 5.15 V; a 2.6 Ah pouch cell was further demonstrated with a reported specific energy of 349 Wh kg^-1^.

Composite electrolytes seek to combine the complementary properties of polymers and inorganic conductors. Ceramic fillers may reduce polymer crystallinity, immobilize anions or create interfacial transport pathways, while the polymer provides flexibility and electrode contact (Shi et al., 2023). The most promising designs move beyond random particle dispersion. Lan et al. (2026), for example, developed continuously perpendicular-aligned ion-conduction pathways in a composite electrolyte and reported 10.2 mS cm^-1^ conductivity at 25 °C. Coin and pouch configurations were operated under very low external pressure, highlighting how controlled electrolyte architecture can link fast transport with mechanical accommodation.

This evolution suggests that composite electrolyte design should be treated as a mesoscale structural problem. Filler orientation, percolation, interfacial chemistry and thickness are often more important than simply increasing the inorganic fraction. The representative characteristics, advantages, and remaining engineering challenges of the major solid electrolyte families are summarized in Table 1.

**TABLE 1:** Comparison of major solid electrolyte families for practical all-solid-state lithium batteries.

Electrolyte family	Representative systems	Main advantages	Key challenges and development focus
Oxides	LLZO, LATP, LLTO	High thermal and electrochemical stability; good mechanical strength	Brittle solid–solid contact, high interfacial resistance, and high-temperature processing; focus on low-temperature densification and interface modification
Sulfides	LGPS, Li_6_PS_5_Cl	High ionic conductivity and good deformability; suitable for cold pressing	Moisture sensitivity, interfacial decomposition, and pressure dependence; focus on stable interphases and low-pressure operation
Halides	Li_3_YCl_6_, Li_3_YBr_6_	Good oxidative stability and compatibility with high-voltage cathodes	Limited stability against Li metal and moisture sensitivity; focus on fluorination, compositional regulation, and protective interlayers
Polymers	PEO-based and fluorinated polymers	Flexible, lightweight, good electrode contact, and easy processing	Low room-temperature conductivity and limited high-voltage stability; focus on reducing crystallinity and improving Li^+^ transport
Composites	Polymer–LLZO, polymer–LATP	Combine inorganic conductivity with polymer flexibility and improved interface contact	Complex transport pathways and filler dispersion; focus on continuous ceramic networks and aligned ion-conduction channels
Emerging systems	Nitrides, multilayer and gradient electrolytes	High design flexibility and potential for specialized interface compatibility	Limited long-term validation and manufacturing maturity; focus on multifunctional interfaces and scalable fabrication

To complement the qualitative comparison in Table 1, Supplementary Table S1 compiles representative quantitative engineering metrics for major solid-electrolyte families and emerging architectures, including room-temperature ionic conductivity, elastic modulus, demonstrated electrolyte thickness, operating stack pressure, and cycling benchmarks. Because these parameters are strongly affected by composition, microstructure, processing history, cell configuration, and test protocol, the reported values are presented as representative literature examples rather than intrinsic constants of individual electrolyte families.

## 4 Interfacial chemo-mechanics and the pressure problem

Interfaces are frequently the dominant source of resistance and failure in solid-state batteries. In a liquid cell, the electrolyte naturally wets newly generated electrode surfaces. In a solid-state cell, loss of physical contact is not spontaneously repaired. Rigid solid–solid contact, interfacial electrochemical reactions and lithium penetration therefore remain central limitations for practical ASSLBs.

At the cathode, anisotropic lattice expansion, particle cracking and interphase growth modify local contact during repeated cycling. Coatings can reduce direct chemical reaction, but a chemically stable coating is useful only when it also permits rapid Li-ion transport. At the lithium side, stripping can generate voids faster than lithium or the surrounding electrolyte can redistribute. Subsequent plating concentrates current at the remaining contact points, accelerating nonuniform deposition. Wan et al. (2023a) therefore emphasized deliberate interface design, while their related analysis proposed critical interphase overpotential as a criterion for suppressing dendrite initiation (Wan et al., 2023b).

External stack pressure is commonly used to compensate for contact loss, but pressure is not a free design variable. Large mechanical fixtures increase pack mass and complexity. Excessive pressure can also promote lithium creep or introduce heterogeneous stress fields. This dual role of pressure is important: moderate compression can improve solid–solid contact, whereas excessive pressure may accelerate lithium creep and generate heterogeneous stress fields that favor localized lithium penetration.

Most importantly, the assumption that a hard ceramic mechanically blocks soft lithium has become increasingly difficult to sustain. Ning et al. (2023) directly investigated dendrite initiation and propagation in solid-state lithium cells. More recent cryogenic electron microscopy and fracture modeling of garnet electrolytes showed lithium filling and mechanically loading crack tips, with hydrostatic stress driving intergranular and transgranular fracture (Zhang et al., 2026). The design objective should therefore be to suppress defects, control electronic leakage, homogenize Li-ion flux and redirect or blunt crack propagation rather than relying solely on electrolyte modulus.

From a mechanical-design perspective, solid electrolytes should therefore be optimized within a property window rather than toward maximum stiffness. In the bulk separator, sufficient modulus is required to preserve geometry and resist compressive deformation under stack pressure, but increasing modulus without simultaneously increasing fracture toughness can amplify sensitivity to pores, grain-boundary flaws and crack-tip lithium penetration. At the Li/electrolyte interface, greater compliance and adhesion improve conformal contact and reduce void formation during stripping, whereas excessive softness or creep can facilitate local lithium extrusion and stress localization. At the cathode side, compliant or graded regions can accommodate active-particle volume changes and reduce delamination, but they must still maintain continuous ion-conduction pathways and dimensional stability.

Accordingly, elastic or shear modulus should not be reported as an isolated mechanical descriptor. Fracture toughness or fracture energy, flaw population, creep/viscoplastic relaxation, interfacial adhesion and pressure-dependent deformation should be considered together. A useful architecture therefore consists of a defect-tolerant and sufficiently strong bulk electrolyte combined with compliant, well-adhered interfaces or gradual mechanical transitions. Stack pressure should likewise be high enough to maintain contact but low enough to avoid excessive lithium creep, particle fracture or heterogeneous stress concentration.

Recent operando and *in situ* characterization methods are increasingly important for distinguishing chemical degradation from mechanically driven contact loss. Operando X-ray tomography provides three-dimensional visualization of buried interfaces and can quantify the evolution of voids, cracks and lithium morphology during cycling without disassembling the cell. Beyond the early observation of coupled void and interphase evolution, recent tomography studies have resolved spatially heterogeneous Li growth and stripping in solid-state architectures and related filament formation to local resistance, pre-existing defects and interface morphology. Such measurements are particularly valuable because stack pressure and temperature can be incorporated as experimental variables rather than treated as uncontrolled boundary conditions.

*In situ* electron microscopy provides complementary information at much smaller length scales. TEM and cryogenic electron microscopy can directly resolve interphase growth, lithium penetration, volume expansion and loss of contact at ceramic/Li interfaces, although electron-beam effects and the simplified geometry of nanoscale cells should be considered when extrapolating to practical batteries. Atomic force microscopy provides another nanoscale route for following SEI formation and lithium nucleation/deposition while simultaneously accessing surface morphology and local mechanical response (He et al., 2024). At the cell level, operando pressiometry, displacement measurements and related *in situ* mechanical techniques can correlate electrochemical reactions with stress accumulation, relaxation and irreversible deformation. The combination of three-dimensional tomography, nanoscale microscopy and cell-level mechanical diagnostics therefore provides a multiscale framework for identifying whether failure originates from chemical interphase growth, void generation, crack propagation or pressure-induced deformation.

Interlayers offer one practical route. Metallic, alloy, polymeric and conversion-type layers can improve lithium wettability, reduce nucleation barriers and produce ion-conductive/electronically insulating interphases. Wang et al. (2024) showed that rational lithium-anode interlayer design can substantially change lithium deposition behavior. Such interfaces should increasingly be evaluated according to long-term chemical evolution, mechanical stability and areal-capacity tolerance rather than initial impedance alone.

## 5 Thin electrolytes and scalable cell manufacturing

A laboratory electrolyte pellet several hundred micrometers thick may be useful for studying intrinsic properties, but it cannot support competitive cell-level energy density. Practical ASSLB manufacturing therefore requires thin electrolyte layers, high cathode active-material fractions, high areal loading and minimal inactive packaging (Zhou et al., 2025). Manufacturing conditions must simultaneously avoid cracking, porosity and chemical contamination.

Several approaches illustrate this transition. Melt infiltration can introduce ion-conducting phases into preformed porous electrode structures and may reduce the need for conventional slurry-based composite fabrication (Xiao et al., 2021). Solvent-free processing is particularly attractive for sulfides because it minimizes drying requirements and solvent–electrolyte reactions. Hu et al. (2024) demonstrated a fusion-bonding strategy for solvent-free fabrication of sulfide electrolyte layers with thicknesses of approximately 25 μm or below, illustrating the importance of processing methods that integrate electrolyte densification with electrode bonding.

Dry co-processing can additionally reduce interface formation steps. Lee et al. (2025) developed a co-rolling dry process that integrated a thin solid electrolyte with a high-loading positive electrode. The reported architecture used an approximately 50 μm electrolyte and a cathode loading near 5 mAh cm^-2^, while retaining more than 80% capacity after 500 cycles under 2 MPa in laboratory cells; pouch-cell demonstrations were also performed at moderate pressure. Such results show why manufacturing architecture now directly determines electrochemical performance rather than simply following materials development.

Cathodes themselves must also become pressure tolerant. Gao et al. (2022) demonstrated that electrode microstructure can be engineered for solid-state operation at relatively low pressure. This is an important shift: instead of treating pressure as an external experimental parameter, pressure dependence should be considered an intrinsic characteristic of the electrolyte–electrode architecture.

Despite these promising demonstrations, scale-up introduces constraints that are weakly represented by laboratory cells. For low-temperature ceramic processing, the central challenges include maintaining phase purity, lithium stoichiometry, densification and low defect density over large areas while avoiding residual porosity and thermal-expansion mismatch during co-processing with electrodes. For dry co-rolling, continuous powder feeding, binder distribution, thickness uniformity, edge cracking, particle fracture and interfacial delamination must be controlled across meter-scale webs; sulfide processing additionally requires stringent atmospheric control because small amounts of moisture can change both chemistry and processability. Melt infiltration faces a different set of limitations, including control of melt viscosity and wetting, complete penetration through thick high-loading electrodes, trapped pores, reactions during heating, solidification shrinkage and sufficiently short processing times for continuous production.

Industrial viability will therefore depend not only on achieving thin electrolytes in individual cells but also on process yield, web-width uniformity, defect detection, materials utilization, dry-room requirements, throughput and compatibility with pouch-cell assembly. Scalable manufacturing should consequently be evaluated using manufacturing-level descriptors such as thickness variation, defect density, processing rate and large-area reproducibility in addition to electrochemical performance.

For future comparisons, electrolyte studies should therefore report at least electrolyte thickness, cathode areal capacity, active-material fraction, operating temperature, applied stack pressure and whether the pressure is actively maintained during cycling. Ionic conductivity measured from thick pellets is no longer sufficient to establish practical relevance.

## 6 Outlook: from record materials to balanced cell design

Solid electrolyte research is entering a stage in which improvements in a single material property provide diminishing practical returns. Conductivities of several inorganic electrolytes are already sufficiently high for battery operation, while new polymer and composite systems continue to narrow the transport gap. The critical challenge is transferring these properties into thin, defect-tolerant and manufacturable cells.

Three directions appear particularly important. First, interfacial design must become mechanically informed. Chemical coatings, artificial interphases and lithiophilic layers should be designed together with stress distribution, void evolution and fracture resistance. Second, low-pressure operation should become a routine benchmark. Demonstrations at tens or hundreds of megapascals may identify promising chemistry but provide limited information regarding deployable battery systems. Recent operation at only a few megapascals—or even near pressure-free conditions—shows that reducing mechanical dependence is increasingly achievable.

Third, electrolyte development should be integrated with manufacturing from the beginning. Low-temperature garnet fabrication, thin sulfide membranes, dry electrode processing and structured composite electrolytes demonstrate that chemistry, mechanics and processing can no longer be optimized independently. Advanced characterization and modeling will be essential for linking local stress, interphase chemistry, ion flux and microstructural evolution across multiple length scales.

A useful practical target is therefore not the “highest-conductivity electrolyte,” but an electrolyte architecture capable of maintaining low resistance at room temperature when fabricated below roughly 50 μm, coupled to cathodes with several mAh cm^-2^ areal capacity and operated under moderate pressure. These values should be regarded as engineering benchmarking directions rather than universal thresholds because the optimum conditions depend strongly on electrode chemistry and cell design. Ultimately, the most successful ASSLBs are likely to emerge from deliberate balancing of ion transport, interfacial stability, mechanical reliability and scalable fabrication rather than from optimization of any one of these characteristics.
